# Is It Definitely Clear That Long-Term Survival after Breast Cancer Surgery Is Not Affected by Anaesthetics?

**DOI:** 10.3390/cancers13143390

**Published:** 2021-07-06

**Authors:** Mats Enlund

**Affiliations:** Centre for Clinical Research, Uppsala University, Central Hospital, SE-72189 Vasteras, Sweden; mats.enlund@regionvastmanland.se; Tel.: +46-21-173775

**Keywords:** anaesthesia, breast cancer, propofol, volatile anaesthetics, survival analysis

## Abstract

**Simple Summary:**

The choice of anaesthetic may affect long-term survival, as suggested in animal studies and in retrospective patient studies. Breast cancer seems to be an exception, according to results from retrospective patient studies. So far this has not been proven in randomised clinical trials. The current state of research is summarised in this overview. The conclusion is that today it seems that the choice of anaesthetic does not play a role in long-term survival after breast cancer surgery.

**Abstract:**

Retrospective studies indicate that cancer survival may be affected by the anaesthetic technique. Propofol seems to be a better choice than volatile anaesthetics, such as sevoflurane. The first two retrospective studies suggested better long-term survival with propofol, but not for breast cancer. Subsequent retrospective studies from Asia indicated the same. When data from seven Swedish hospitals were analysed, including 6305 breast cancer patients, different analyses gave different results, from a non-significant difference in survival to a remarkably large difference in favour of propofol, an illustration of the innate weakness in the retrospective design. The largest randomised clinical trial, registered on clinicaltrial.gov, with survival as an outcome is the Cancer and Anesthesia study. Patients are here randomised to propofol or sevoflurane. The inclusion of patients with breast cancer was completed in autumn 2017. Delayed by the pandemic, one-year survival data for the cohort were presented in November 2020. Due to the extremely good short-term survival for breast cancer, one-year survival is of less interest for this disease. As the inclusions took almost five years, there was also a trend to observe. Unsurprisingly, no difference was found in one-year survival between the two groups, and the trend indicated no difference either.

## 1. Introduction

We have learned from pre-clinical studies on animals and human cell-lines, that general anaesthetics act differently on the immune system [1,2,3,4,5,6]. Volatile anaesthetics, such as sevoflurane, the most commonly used anaesthetic for anaesthesia maintenance, are pro-inflammatory and add an inflammatory urge to the innate response started by the surgical insult [7,8,9]. This is negative for the immune defence against cancer cells, since the surgical insult promotes tumour metastasis [10]. Pro-inflammatory mediators, such as interleukin 1 and 6 and tissue-necrosis factor alpha, get triggered, and the inflammatory process will wind up. Clinical studies have indicated similar effects in patients [11,12]. In contrast, propofol seems to have an anti-inflammatory effect, which theoretically can be good for healing from the cancer, counter-balancing the surgery-driven inflammatory urge [13,14,15].

Volatile anaesthetics are genotoxic, as indicated when measuring increased levels of the marker for DNA-damage, sister-chromatid exchange in leukocytes or with the micronucleus assay after drug exposure [16,17,18,19]. Such a mechanism could potentially adversely affect the patient’s survival after cancer surgery. The less studied propofol seems not to be genotoxic [20].

Hypoxia-inducible factor (HIF) is a transcription factor that stimulates and regulates cancer cells’ adaptation to an unfavourable environment with hypoxia, acidosis, and lack of nutrients, which develops centrally in a growing solid tumour. Our knowledge about HIF comes a great deal from the groups led by Prof. Kazuhiko Fukuda, Kyoto, Japan and Prof. Daqing Ma in London, UK [21,22]. They show that HIF is up-regulated when the cancer cells are exposed to a volatile anaesthetic [23]. Then, the cancer cells, that are escaped locally or to the blood stream during surgery, would have a better chance to survive as a local recurrence or as metastases. Propofol seems to down-regulate this transcription factor [24].

Striking laboratory results from breast cancer have been published from several groups, not least from Prof. Donal Buggy’s group in Dublin, Ireland. e.g., natural killer cell activity was measured and compared between women anaesthetised with either a sevoflurane-opioid combination or with propofol combined with a paravertebral block [25]. The natural killer cell activity was higher in the latter group. Moreover, cell cancer apoptosis was higher in the propofol/paravertebral group [26]. Also, propofol was shown to have a more favourable effect on vascular endothelial growth factor C and transforming growth factor beta [27]. These findings indicate an advantage with propofol, irrespective if opioids would be replaced by regional anaesthesia or not. The impact of morphine on cancer cure is questioned with indications of an immunosuppressive effect [28,29,30], but also the opposite [31,32].

The question is, if such findings, as opposite actions on the immune system, the DNA, and the HIF, translate to longer survival? To investigate the current state of knowledge, a simple search was conducted on PubMed in January this year with the following search terms: sevoflurane/desflurane or propofol and cancer/neoplasms and outcome/survival.

## 2. Retrospective Patient Studies

**Figure 1:**
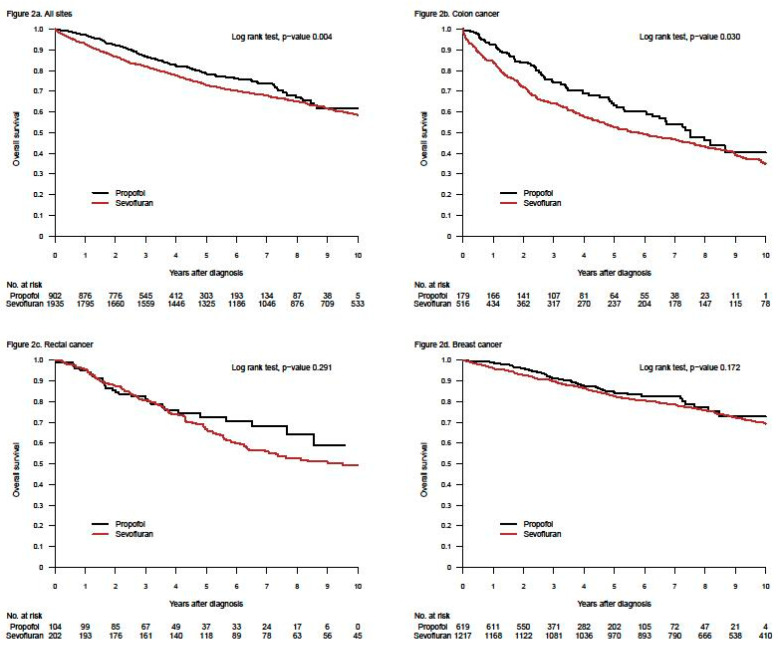
Kaplan–Meier curves for survival after propofol- or sevoflurane-based anaesthesia for three different cancer locations (all sites together = a; colon = b, rectum = c, and breast = d). The upper left graph was published in reference [33], Upsala Journal of Medical Sciences, Taylor & Francis (Creative Commons Attribution License).

In this new research field, the first retrospective study, investigating the impact of general anaesthetics on long-term survival directly comparing propofol with sevoflurane, was published in 2014 [33]. Data from 2838 patients, registered for surgery for breast-, colon-, or rectal cancers at our hospital, were analysed and record-linked to regional clinical quality registers. Cumulative 1- and 5-year overall survival rates were assessed using the Kaplan–Meier method, and estimates were compared between patients given propofol (*n* = 903) or sevoflurane (*n* = 1935) (Figure 1). In a second step, Cox proportional hazard models were calculated to assess the risk of death adjusted for potential effect modifiers and confounders. The absolute difference in overall 1- and 5-yr survival rates for the three cancer sites combined was 4.7 percentage points, (*p* = 0.004) and 5.6 percentage points (*p* < 0.001), respectively, in favour of propofol. However, after adjustment for unequal distribution of confounders, the observed differences were not statistically significant in this study; hazard ratio (HR) for propofol vs. sevoflurane was 0.85 (95% CI: 0.72–1.00; *p* = 0.051).

Later, the hypothesis was strengthened in a second retrospective study from London, UK [34]. Several different cancers were examined there, including breast cancer. For all patients, who received propofol in the cited study, the 1-year survival rate was 94.1% (95% confidence interval (CI), 93.3 to 94.8), whereas for the patients in the inhalational cohort (mainly sevoflurane; personal communication with first author, T. Wigmore, May 2016) it was 87.9% (95% CI, 86.7 to 89.1), (*p* < 0.001). The mortality rate during a median follow-up period of 2.7 years was 13.6% (504 of 3714) in the propofol cohort and 24% (796 of 3316) in the sevoflurane cohort, and inhalational anaesthesia was associated with a HR of 1.59 (95% CI, 1.30 to 1.95) for death on univariate analysis and 1.46 (95% CI, 1.29 to 1.66) after multivariable analysis of known confounders in a propensity score match.

More similar studies have been published, some with overall survival at different time points as the main outcome, some studying the duration of recurrence free survival [35,36,37,38,39,40,41,42,43,44,45,46,47,48,49,50,51,52,53,54,55,56]. The number of included patients in studies with results that do not support the hypothesis [33,37,38,42,43,44,45,46,47,48,49,50] and studies supporting it [34,35,36,39,40,41,51,52,53,54,55,56], are quite equal. If anything, there are more patients in studies without support for the hypothesis. Four meta-analyses, however, all conclude a survival gain with propofol, the latest was from 2020 [57,58,59,60] (Table 1 and Table 2). That said, some of these retrospective studies are small, some extremely so, making the already dubious value of retrospective design even more uncertain. It should also be noted that the largest retrospective study to date (*n* = 6305), which examined overall survival from breast cancer, is not included in any of the tables due to its dubious results [61].

**Figure 2:**
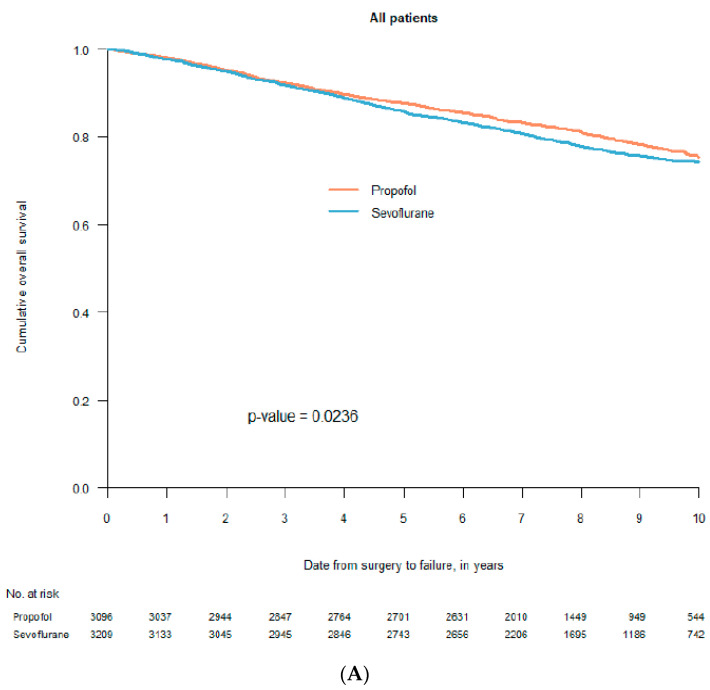

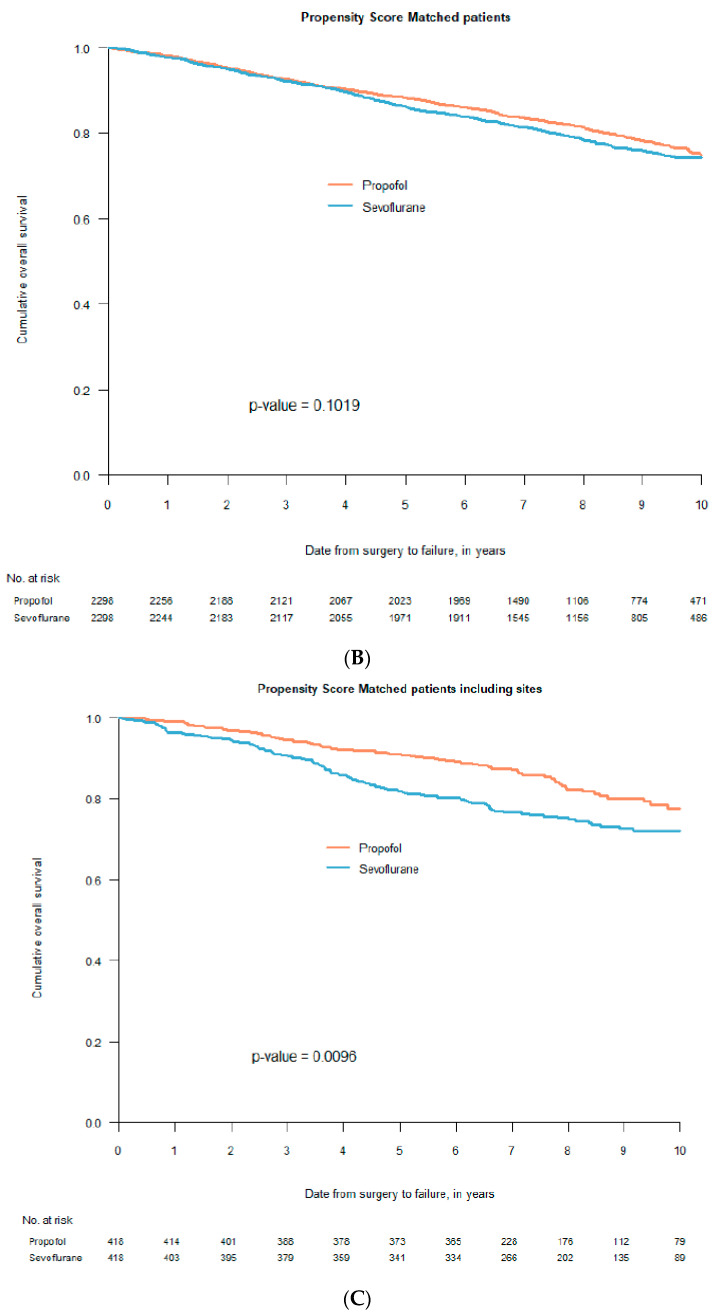
Kaplan–Meier curves for survival after propofol- or sevoflurane-based anaesthesia. Graph (**A**) includes all 6305 patients before statistical adjustments; graph (**B**) includes 2298 pairs after propensity score match without the seven participating clinics included as a co-factor in the statistical analysis; graph (**C**) includes 418 pairs after propensity score match with the seven participating clinics included in the statistical analysis (with permission from Acta Anaesthesiologica Scandinavica, Wiley; reference [61]).

What did not specifically appear in the first two retrospective studies was that survival after breast cancer surgery was not affected by the choice of anaesthetic [33,34]. Other cancers explained the found differences (Figure 1 and personal communication with Tim Wigmore, December 2019). This was not obvious in the publications (in our case a decision made by the editor). Moreover, three retrospective studies from Asia, specifically investigating breast cancer, indicated no difference in long-term survival between patients anaesthetised with propofol or sevoflurane [37,42,43]. The largest study of the three included 5331 patients [42], in all 8952, of whom a third of the patients were used in propensity score match. A fourth study, measuring freedom from recurrence within one year, indicated the same—no difference between the two anaesthetics [48]. Later, data from seven Swedish hospitals, including 6305 breast cancer patients, were analysed in a new retrospective study [61]. Two of the participating clinics used propofol, two used sevoflurane and the other three used both drugs alternately in a pseudo-random way. Different statistical analyses gave different results in overall survival, from a non-significant difference to a remarkably large—hard to believe in—difference, favouring propofol (Figure 2). This was an illustration of the innate weakness of the retrospective design.

National register-based studies with a retrospective analysis of prospectively collected data may be a step up for the evidence base and offer not only larger numbers and thereby better precision, but also the possibility to adjust for more confounders and effect modifiers. Two recent national register-based studies on patients with gastro-intestinal cancer from Japan and colorectal cancer from Denmark, including 196,303 and 11,650 patients, respectively, indicated however no difference between propofol- or inhalational maintained anaesthesia [62,63]. Both studies were published after the four meta-analyses. It must be said however, that such an impressive number of patients included in a retrospectives study does not automatically mean that the truth is found. Even in the large Japanese study, doubts were present. The group of patients given an inhalant anaesthetic was almost six times larger than the group given propofol. More importantly, the authors could only identify patients who died in the hospital in which they had their cancer surgery, i.e., patients who died at home or elsewhere were lost for follow up. Moreover, the postoperative follow-up period was short with a median of just over two years.

Depending on the amount of patient characteristics available in different retrospective studies, the possibilities to statistically adjusted for the impact from confounders and effect modifiers varies. Selection bias is an elusive mechanism. Different decisions from anaesthetists, who can evaluate patients differently and thus consider different anaesthetic methods or use different drugs for patients, are often a source of non-adjustable bias, unless a certain hospital has a standard regimen as described for some hospitals in reference [61].

## 3. Randomised Clinical Trials (RCTs)

Results from retrospective studies can be conducted faster and cheaper than randomised clinical trials (RCTs), but they can only indicate the direction of the evidence—“it may be like this”—and they can provide a basis for a proper analysis of statistical power ahead of an RCT. From our first results and from national Swedish data, we calculated that we would need 1650 patients with breast cancer to have a chance to show a difference of at least five percentage points in five-year survival between patients anesthetized with propofol or sevoflurane for surgical removal of the cancer in an RCT, assuming an alpha error of less than 5% and a beta error of less than 20% [33]. The study is named the Cancer and Anaesthesia study (CAN), (EudraCT, 2013-002380-25 and ClinicalTrials.gov, NCT01975064) [64]. We received approval for a margin of up to 8% and used a large part of this to include a total of 1750 patients with breast cancer in the CAN study, of whom 1702 were analysed. In two other arms, we include patients with colo-rectal cancer. Here, the inclusions are still on-going, aiming for 3000 and 2728 patients, respectively.

**Figure 3:**
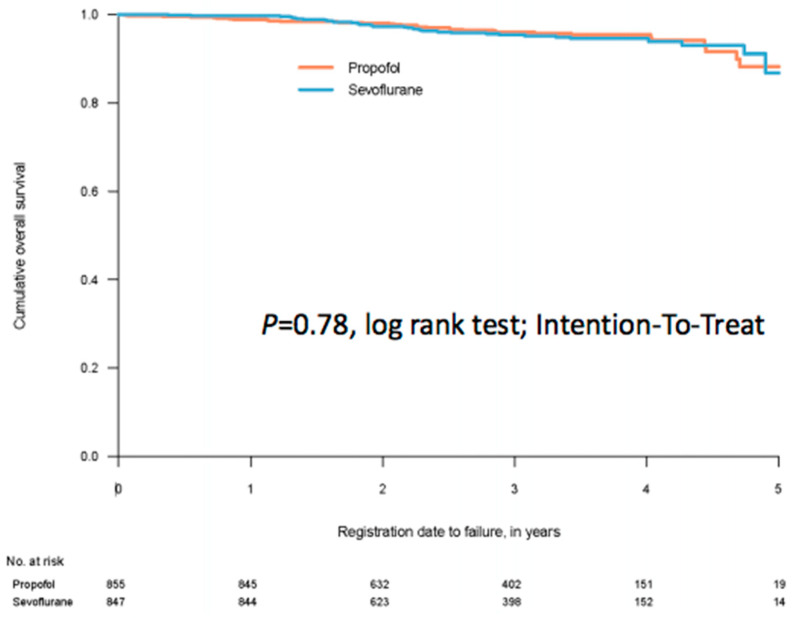
A Kaplan–Meier curve for survival after anaesthesia for breast cancer surgery on 1702 patients, of which 855 were randomised to propofol for anaesthesia maintenance and 847 patients were randomised to sevoflurane for anaesthesia maintenance. The time for follow up was minimum one year, median 2.66 years. From reference [65].

With almost no deaths within the first postoperative year after breast cancer surgery, one-year survival is of limited or no interest. When working the project plan, we did not separate outcomes for the three cohorts. Thus, one-year survival was chosen as a secondary outcome also for breast cancer. While waiting for five-year follow up (in late 2022) [64] we recently analysed one-year survival for the breast cohort (severely delayed by the pandemic) [65]. Since the inclusions took almost five year to complete, we supplemented the one-year result with a trend over the data collection period. We could not demonstrate any meaningful difference between the two groups (Figure 3).

An interesting RCT, conducted in Switzerland, investigated the count of circulating tumour cells and the association between natural killer cell activity and tumour cell count in 210 patients with breast cancer, assigned to either propofol or sevoflurane anaesthesia [66]. The count of circulating tumour cells is considered a prognostic marker. No difference was observed in the count of circulating tumour cells between the two groups, and there was no association between natural killer cell activity and tumour cell count. The study was obviously not powered for studying survival.

It is sparse with RCTs investigating the possible impact of anaesthetic drugs on long-term survival after cancer surgery in general and breast cancer in particular. In the largest study so far, survival was replaced by recurrence rate, which may be regarded as a surrogate marker for survival [67]. In the cited study 2132 women were assigned to either propofol and paravertebral block or sevoflurane and opioid for breast cancer surgery. The median follow-up time was three years, none less than two years. The recurrence rates were identical in the two groups, 10%. For obvious reasons, it is not possible to separate the effects of propofol vs. sevoflurane and paravertebral block vs. opioids in the study. Consequently, it is impossible to determine whether the lack of effect of the perioperative care of the breast cancer patients was due to an actual absence of effect, or whether the combination of measures masked an actual effect of any of the constituent components.

Recurrence-free survival was followed up after two years observation time in a study, in which the authors first measured the impact of a sevoflurane-based anaesthetic and a propofol-remifentanil anaesthetic on the release of VEGF-C and TGF-β [68]. These factors promote angiogenesis, and they have a theoretical interest in the development of a cancer. The total number of patients was 80, which was enough for to demonstrate a significant inhibition of the two promoters of angiogenesis in the propofol-remifentanil group, but not in the sevoflurane group. In the light of the numbers needed in the aforementioned RCTs, 80 patients are far from a number that is meaningful for studying recurrence-free survival, or for that matter overall survival.

Thus, two RCTs with accurate statistical power have been reported so far [65,67]. Albeit with some limitations, both indicate that there is no effect of anaesthetics on survival after breast cancer surgery.

## 4. Discussion

Can just a few hours of exposure to an anaesthetic really affect survival for several years to come? The hypothesis, that the choice of anaesthetic is important for long-term survival after a cancer operation, is at first glance almost bizarre. However, the theoretical basis is compelling, and when supported by a large number of retrospective studies and even meta-analyses, the idea becomes convincingly attractive.

Obviously, different cancers may have varying properties to cause local recurrence or metastases, i.e., tumour biology differs, and the activation of adrenergic-inflammatory stimulus may differ, e.g., triple-negative breast cancer is associated with a stronger adrenergic response than other breast cancers [69], and it is close at hand to suspect variations in adrenergic response between different other cancers. Therefore, if anaesthetic drugs modify tumour biology, the resulting effect may vary between different cancers. The duration of surgical procedures and the exposure to anaesthetic drugs vary as well, which could play a role for cancer recurrence and mortality. If the results of the many positive retrospective studies really mirror reality, indicating the truth, it should be noted that breast cancer surgery is a relatively short procedure. Thus, the exposure to a “good” or a “bad” anaesthetic is relatively short, which may have a meaning. Technical aspects of the surgical procedures also vary. A superficial tumour, like a breast cancer, may be easier to manage with lower risk of cancer cell dissemination. Thus, the current knowledge of anaesthetic impact on breast cancer survival should not be automatically extrapolated to other cancer types.

At the top of the pyramid of evidence-based medicine are well-dimensioned and well-executed RCTs, or rather a meta-analysis of such studies is placed at the top, resting on the RCTs the next step below. Therefore, we wish more RCTs in this field, and preferably with survival as the ultimate outcome. Given the challenge in funding and the hard work during a long time span, we cannot however expect many RCTs. Since retrospective studies have strong limitations [61], the second best to RCTs would be national quality registers for retrospective analyses of prospectively collected data [62,63]. This author has the result of such a register-based study for breast cancer submitted.

## 5. Conclusions

The current state of knowledge is, that it does not seem to matter which anaesthetic is used to maintain anaesthesia during breast cancer surgery. The conclusion is based on the results of five retrospective studies and two RCTs where breast cancer was studied, albeit with some limitations for the latter—only one-year survival available so far in one case and only recurrence rate observed instead of survival in the other case. The result from one of the RCTs regarding long-term survival (minimum five years) after breast cancer surgery is expected in late 2022 or early 2023, and the result from a national register-based study will come later this year.

## Figures and Tables

**Table 1:** Retrospective patient studies *without* a significantly longer survival for patients given propofol compared with patients given an inhalant anaesthetic. PSM = propensity score match.

Journal	Year	First Author	Cancer Localisation	Total Number of Patients
*Upsala J. Med. Sci.* [33]	2014	Enlund, M.	Breast, colo-rectal	2838
*Oncotarget* [37]	2017	Kim, M.H.	Breast	2645
*Cancer Control.* [38]	2018	Oh, T.K.	Lung	943 (392 in PSM)
*Anesthesiology* [42]	2019	Yoo, S.	Breast	5331 (1766 in PSM)
*PLoS ONE* [43]	2019	Huang, Y.-H.	Breast	976 (888)
*Acta Anaesthesiol. Scand.* [44]	2019	Oh, T.K.	Gastric	4607 (1538 in PSM)
*BMC Anesthesiol.* [45]	2019	Hong, B.	Mixed locations	1458
*J. Neurosurg. Anesthesiol.* [46]	2019	Dong, J.	Brain (glioma)	294
*Sci. Rep.* [47]	2020	Grau, S.J.	Brain (glioma)	158 (158 in PSM)
*J. Anesth.* [48]	2020	Shiono, S.	Breast	1026
*Neurosurg. Rev.* [49]	2020	Schmoch, T.	Brain (glioblastom)	144
*Dan. Med. J.* [50]	2020	Hasselager	Colorectal	534
Total number of patients				18,324

**Table 2:** Retrospective patient studies *with* a difference in survival for patients given propofol compared with patients given an inhalant anaesthetic. PSM = propensity score match.

Journal	Year	First Author	Cancer Localisation	Total Number of Patients
*Anesthesiology* [34]	2016	Wigmore, T.	Mixed cancers	7030
*Korean J. Anestesiol.* [35]	2016	Lee, J.H.	Breast	325
*Sci. Rep.* [36]	2017	Jun, I.J.	Esophagus	922
*Onco. Targets Ther.* [39]	2018	Zheng, X.	Gastric	2856 (897 in PSM)
*Anesthesiology* [40]	2018	Wu, Z.F.	Colon	1363 (1158 in PSM)
*Br. J. Anaesth.* [41]	2019	Lai, H.-C.	Liver	944 (670 in PSM)
*PLoS ONE* [51]	2020	Lai, H.-C.	Pancreas	140 (116 in PSM)
*PLoS ONE* [52]	2020	Lai, H.-C.	Prostate	631 (528 in PSM)
*Medicine (Baltim.)* [53]	2020	Huang, N.C.	Gastric	408 (334 in PSM)
*Med. Princ. Prac.* [54]	2020	Koo, B.-W.	Liver	535
*BMC Anesthesiol.* [55]	2020	Meng, X.Y.	Liver	1513
*Surg. Today* [56]	2021	Hayasaka, K.	Lung	230
Total number of patients				16,897
